# Type 1 Collagen/Poly-L-Lactic Acid Biocomposite Augmentation of a Nontraumatic Symptomatic Complete Rotator Cuff Tear

**DOI:** 10.31486/toj.25.0068

**Published:** 2026

**Authors:** J. Heath Wilder, Jonathan Willard, Eden Schoofs, Brianna Bennett, Graylin Jacobs, Joseph Laurent, Deryk Jones

**Affiliations:** ^1^ Ochsner Andrews Sports Medicine Institute, Ochsner Clinic Foundation, New Orleans, LA; ^2^ Department of Emergency Medicine, Ochsner Clinic Foundation, New Orleans, LA; ^3^ The University of Queensland Medical School, Ochsner Clinical School, New Orleans, LA

**Keywords:** *Collagen type 1*, *dermal fillers*, *rotator cuff*, *rotator cuff injuries*, *suture anchors*, *tissue scaffolds*

## Abstract

**Background:**

Treatment modalities for rotator cuff tears are continuously evolving. Augmentation of rotator cuff repair with biocomposite implants with bioinductive properties such as collagen scaffolds, dermal grafts, and synthetic materials are increasingly being used to ensure repair integrity. Ideally, an implant should provide added structural integrity as well as biologic components that augment the final repair tissue quality.

**Case Report:**

A 69-year-old male underwent arthroscopic rotator cuff repair augmented with a BioBrace Reinforced BioInductive Implant (CONMED Corporation), a biocomposite implant containing type 1 bovine collagen with poly-L-lactic acid. With physical therapy, the patient's periscapular and rotator cuff strengthening had progressed by 8 weeks postoperatively, and full return to activities began at 6 months postoperatively. Postoperative magnetic resonance imaging at 1 year demonstrated complete healing of the rotator cuff repair with intact footprint on coronal and sagittal images.

**Conclusion:**

This biocomposite implant provides an encouraging option for use in patients with poor tissue quality or tear patterns that are concerning for possible postoperative retear.

## INTRODUCTION

Rotator cuff injuries—one of the most common upper extremity conditions seen by orthopedic surgeons—can range from tendinopathy to complete tears, so clinical manifestation varies considerably. Some patients are asymptomatic, but, more commonly, patients present with decreased range of motion, especially noticeable when performing overhead tasks, and shoulder pain that can radiate into the deltoid region.^1-3^ Rotator cuff pathology is particularly prevalent among older patients. Dang and Davies reported that asymptomatic cuff abnormalities are seen in 30% of patients 60 years or older and in 62% of patients 80 years or older.^1^ Minagawa et al performed health care checkups of “locomotive organs” for 664 patients in a village in Japan and found 147 patients (22.1%) with full-thickness rotator cuff tears; 34.7% of the tears were symptomatic, and villagers ≥60 years of age accounted for approximately 78% of the tears.^4^

Treatment options described in the literature are largely dependent on the patient's symptoms, functionality, age, the extent of the tear, and the surrounding muscle quality.^5,6^ The size of untreated tears may increase, and the torn rotator cuff muscle may atrophy.^7^ Most patients are initially managed nonoperatively;^8^ however, Boorman et al reported that approximately 25% of a conservatively treated cohort of 93 patients with rotator cuff tears progressed to surgical intervention during a 5-year period.^9^

Technology advances have transformed surgical techniques for rotator cuff repair, and good clinical outcomes are common. However, potential complications include stiffness, retear, infection, and pain.^10,11^ Biocomposite implants with bioinductive properties, including collagen scaffolds, dermal grafts, and synthetic materials, are increasingly being used to improve the integrity of rotator cuff repairs.^12,13^ The BioBrace Reinforced BioInductive Implant (CONMED Corporation) is a biocomposite scaffold composed of porous type 1 bovine collagen fibers reinforced with bioresorbable poly-L-lactic acid microfilaments.^14^ The implant allows for induction, maturation, and remodeling of new host tissue while providing load-sharing strength at time 0 of implantation.^15^

We present a case of symptomatic rotator cuff repair using a BioBrace implant.

## CASE REPORT

A 69-year-old male presented to clinic with long-standing left shoulder pain and weakness for 1 year with acute worsening. Atrophy of the left shoulder was noted on physical examination; Neer, Hawkins-Kennedy, Speed, and cross-arm adduction tests were positive. The patient reported pain with forced abduction and range of motion (ROM); no limitations in ROM were noted in the opposite shoulder.

**Figure 1.**
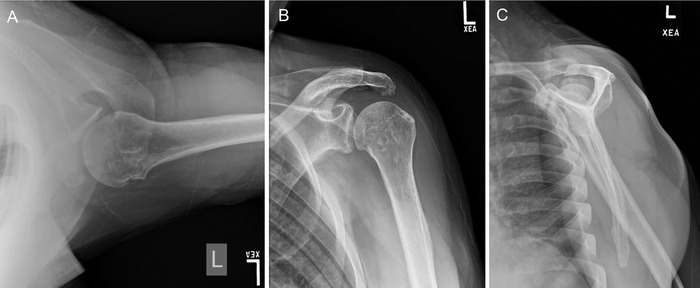
Preoperative radiographs in (A) axillary view, (B) anterior-posterior Grashey view, and (C) scapular Y view show cystic changes of the greater and lesser tuberosities and subacromial enthesopathic spurring consistent with rotator cuff pathology.

**Figure 2.**
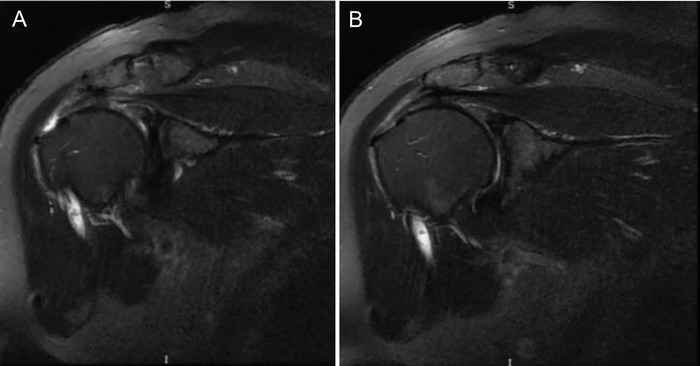
Preoperative magnetic resonance coronal T2 images show (A) supraspinatus full tear and (B) infraspinatus partial tear.

Shoulder radiographs displayed cystic changes in the greater and lesser tuberosity of the glenohumeral joint, as well as subacromial enthesopathic spurring with a type 3 acromion process consistent with rotator cuff pathology (Figure 1). Magnetic resonance imaging (MRI) of the left shoulder showed a 1.6 × 1.3-cm full-thickness defect at the supraspinatus footprint with undersurface irregularity and partial thickness involvement of the infraspinatus (Figure 2), as well as partial tearing at the leading edge of the subscapularis tendon, proximal biceps tendon tearing, and acromioclavicular hypertrophy. Articular cartilage in the glenohumeral joint was intact.

The patient's preoperative Single Assessment Numeric Evaluation (SANE) score was 25/100, consistent with low functional level and substantial symptomatology (Table).^16^ The patient's preoperative EuroQol-5 Dimension, 5-Level (EQ-5D-5L) questionnaire score was 70, also consistent with poor functional level.^17^

**Table.** Patient-Reported Outcome Measures

Time Point	SANE^a^	EQ-5D-5L^b^	Stability^c^	ASES^d^	PSF-12^e^	MSF-12^e^
Baseline	25	70				
6 weeks postoperatively	30	50	0	37.18	35.58	66.87
3 months postoperatively	75	65	3	40.38	40.64	51.51
6 months postoperatively	85	75	0	95.51	51.38	61.65
12 months postoperatively	98	80	0	99.36	54.84	59.78

^a^The Single Assessment Numeric Evaluation (SANE) rates current function in an injured body part and is scored from 0 to 100, with 100 representing normal function.^16^

^b^The EuroQol-5 Dimension, 5-Level (EQ-5D-5L) assesses mobility, self-care, usual activities, pain/discomfort, and anxiety/depression and is scored from 0 to 100, with 100 representing normal function.^17^

^c^Shoulder stability was subjectively reported by the patient on a scale of 0 to 10, with 10 representing normal function.^18^

^d^The American Shoulder and Elbow Surgeons (ASES) Standardized Shoulder Assessment Form assesses patient-reported shoulder pain and dysfunction on a scale 0 to 100, with 100 representing normal function.^19^

^e^The PSF-12 (physical health) and MSF-12 (mental health) are assessments from the 12-Item Short Form Health Survey and are scored on a scale of 0 to 100, with 100 representing normal function.^20^

The treatment decision was to proceed with arthroscopic rotator cuff repair, distal clavicle excision, biceps tenodesis, subscapularis repair, and augmentation with a BioBrace implant if tissue quality or tear pattern was deemed suboptimal for maximum healing potential.

**Figure 3.**
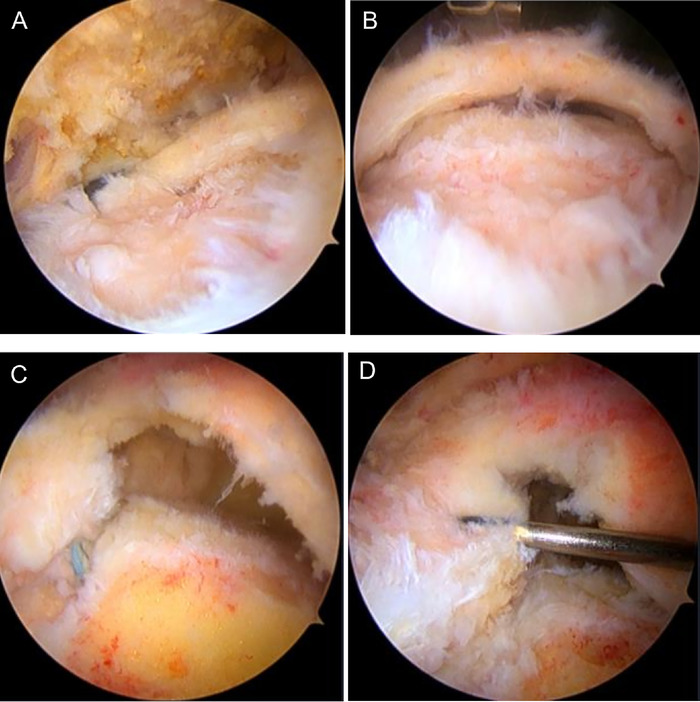
Arthroscopic views show (A and B) retracted crescent-shaped tear with moderate atrophy and larger-than-expected size and (C and D) mobilization prior to fixation.

Diagnostic arthroscopy revealed a >50% tear of the biceps tendon, a full-thickness 4.5-cm crescent-shaped tear of the supraspinatus and infraspinatus tendons with retraction to the glenoid, and muscle atrophy and scarring (Figures 3A and 3B).

A tear in the leading edge of the subscapularis tendon was repaired arthroscopically with a triple-loaded suture anchor placed through an anterior superior portal (Healix Advance, Mitek). Prior to subscapularis repair, the biceps tendon was controlled with a luggage tag suture and tenotomy.

The rotator cuff was mobilized with standard releases but demonstrated initial retraction to the glenoid level and moderate atrophy (Figures 3C and 3D). Because of the level of retraction, the tissue quality, and the size of the tear, a double-row repair was performed with concomitant BioBrace implant augmentation. Two medial double-loaded suture tape anchors (Healix Advance Anchors with Permatape, Johnson and Johnson Medical, Inc) were placed along the medial aspect of the prepared footprint; all 4 suture limbs and all 4 tape limbs were passed through the mobilized supraspinatus and infraspinatus tissues medially. The sutures were tied with a modified Roeder knot, and the remaining suture material was cut.

**Figure 4.**
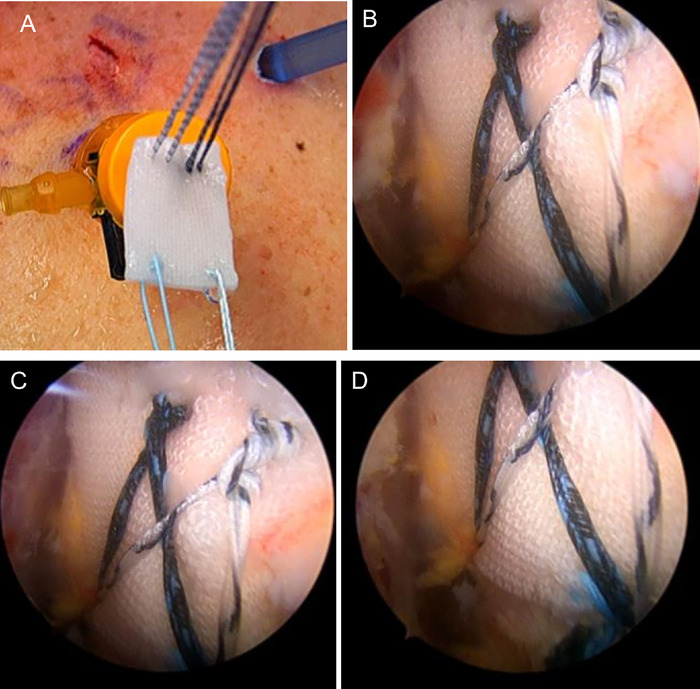
(A) The BioBrace Reinforced BioInductive Implant (CONMED Corporation) is pictured prior to delivery through an 8-mm cannula, with suture tapes passed medially and lateral Mason-Allen sutures. Arthroscopic views show (B and C) the BioBrace implant fixed medially with tapes and (D) stabilized medially with tapes and lateral fixation with knotless anchors using a bridge construct.

The BioBrace was prepared prior to implantation by placing 2 lateral row #2 DYNACORD sutures in modified Mason-Allen fashion. The suture tapes were passed through the medial aspect of the implant in simple, noninterlocking fashion outside the joint space (Figure 4A); a standard knot-tying device was used to shuttle the BioBrace implant through an 8-mm cannula. The tapes were tied medially to stabilize the BioBrace implant and then used to create a bridge construct (Figures 4B and 4C). The mobilized tapes and previously placed #2 DYNACORD sutures were passed sequentially through two 5.5-mm Healix Advance Knotless Anchors (DePuy Synthes), starting from anterior to posterior. The final double-row repair with the BioBrace implant was stable (Figure 4D).

A subpectoral biceps tenodesis was performed through a 2-cm anterior incision with the onlay technique and suture anchor fixation using a double-loaded GRYPHON suture anchor (DePuy Synthes). Standard closure was performed. The patient was placed in a sling with a neutral abduction pillow (Breg, Inc).

**Figure 5.**
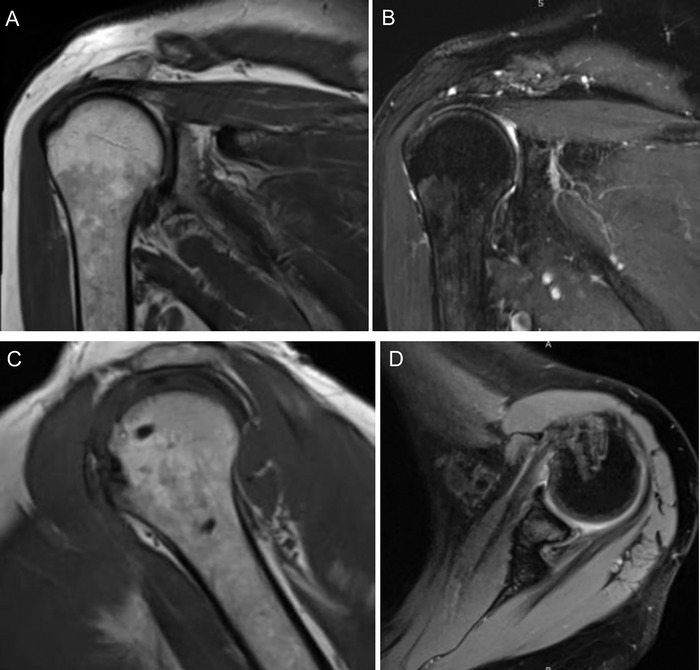
One-year postoperative magnetic resonance (A) coronal T1, (B) coronal T2, and (C) sagittal T1 images show complete healing of the rotator cuff repair. (D) One-year postoperative magnetic resonance axial T2 image shows subscapularis repair.

The patient started physical therapy 2 days after surgery: ROM with external rotation at the side to 30°, abduction to 60°, and forward flexion to 90° passively. Codman pendulum exercises were initiated 5 days postoperatively, and the abduction pillow was discontinued at 4 weeks. Full mobilization and full active-assisted and passive ROM of the extremity were allowed at 6 weeks. Periscapular and rotator cuff strengthening had progressed by 8 weeks postoperatively, and full return to activities began at 6 months postoperatively. Postoperative MRI imaging at 1 year demonstrated complete healing of the rotator cuff repair with intact footprint on coronal and sagittal images, as well as maintained subscapularis repair on axial images (Figure 5). Postoperative patient-reported outcome measures^16-20^ collected at 6 weeks, 3 months, 6 months, and 12 months demonstrated substantial improvement in pain and overall function, while subjective shoulder stability scores remained unchanged (Table).

## DISCUSSION

Chronic shoulder pain is a prevalent societal burden, only trailing chronic knee pain as the most reported musculoskeletal pathology in the United States.^21^ A large percentage of reported shoulder pain is associated with rotator cuff disease, which can be difficult to follow and predict because of a variety of factors, including age, type of tear, and involvement of multiple tendons.^22^ While rotator cuff tears are typically described as acute or chronic, the literature also describes acute-on-chronic tears as pain that was previously manageable but suddenly worsens.^23^ Patients >40 years old are most at risk for rotator cuff tears and may experience symptoms such as pain with lifting or movement, weakness, or crepitus.^24^

Repetitive overhead movements in daily living activities increase the risk of injury, as do repetitive overhead movements in sports, particularly baseball and tennis.^3^ These movements not only can lead to rotator cuff injury but also to subacromial impingement, glenohumeral instability, and acromioclavicular joint arthritis, most commonly in the nondominant shoulder.^25^ Diagnosing rotator cuff injuries involves a variety of tests and measures. Initial assessment requires a full history and physical, with diagnostic imaging in select circumstances.^26^ Accurate preoperative diagnosis is classically achieved with arthrography and arthroscopy, although MRI and ultrasound are the most used techniques.^27,28^

Conservative nonoperative management is the initial treatment modality for rotator cuff tears. Compared to surgery, physiotherapy is less likely to lead to complications and is a less expensive option.^8^ Physiotherapy commonly involves restoring ROM and motor control, increasing strength, and minimizing stiffness.^27^ Kukkonen et al compared the effectiveness of physiotherapy, acromioplasty, and rotator cuff repair for nontraumatic rotator cuff tears and found that conservative treatment is a reasonable option for initial treatment of isolated, symptomatic, nontraumatic, supraspinatus tears in older patients.^29^ Other conservative modalities include nonsteroidal anti-inflammatory drugs, corticosteroid injections, and platelet-rich plasma injections. A randomized trial of 96 patients showed that the combination of physiotherapy and corticosteroid injections was the most effective conservative treatment for full-thickness rotator cuff tears.^30^

When conservative measures fail, surgical treatment is the next step in managing rotator cuff tears. Surgical technique is determined by the size, thickness, and location of the tear.^1^ Bedi et al described massive tears based on their size, involvement of multiple tendons, or the area affected.^31^ While the Bedi et al definition has great variation, massive tears account for 10% to 40% of rotator cuff tears and often require additional techniques such as patch augmentation, defined here as implant-based scaffold augmentation, as used in our case.^31,32^

In a 2022 review, Cobb et al reported that short-term studies showed implant-based scaffold augmentation had great potential to improve the success rate of rotator cuff repair.^33^ Chalmers and Tashjian discussed different implant-based scaffold augmentation types and highlighted that some absorbable, purely biologic implants do not offer much value.^34^ Cai et al reported that augmentation with absorbable, purely biologic implants can have limited effectiveness because of adverse events such as allergic reaction and poor tissue quality, leading to higher retear rates.^35^ Biocomposite collagen implant augmentation seems to be a safe option for rotator cuff repair and may reduce the risk of retear; however, additional studies are needed, as purely biologic implants historically lack structural strength at the time of implantation.^15,36^ Dermal allografts have the potential for promising biomechanical, structural, and functional outcomes in rotator cuff repair, especially in patients with a diminished chance of postoperative healing.^37^ Gatot et al described a single lateral row implant-based scaffold augmentation technique using human dermal allograft as an appropriate and effective method in addressing a degenerate tendon that may be associated with small-sized tears.^38^ Increased operating time, cost, and need for additional surgical assistance should be considered for this method.^39^ Dermal allografts provide structural strength at the time of implantation but have allograft tissue risks, such as immune response, and do not incorporate into the tissue postoperatively.^15^

The BioBrace implant is a highly porous scaffold composed of type 1 bovine collagen with poly-L-lactic acid bioresorbable microfilaments that provides strength for 1.5 years following implantation.^14,15^ This timeline is consistent with the senior author's clinical experience and intraoperative observations. The BioBrace implant characteristics enhance repair healing by combining the strengths of both biocomposite implants with bioinductive properties and dermal allografts, allowing for induction, maturation, and remodeling of new host tissue while providing load-sharing strength that theoretically can prevent gapping and retears by increasing the thickness of the tendon over time while providing strength to protect the repair at the time of implantation.^15,39^ The BioBrace implant has been used to repair tendon injuries other than rotator cuff tears. Le and McMillan used the BioBrace in a distal biceps tendon rupture repair and reported that the BioBrace provides strength at the time of implantation while inducing new tissue growth during the resorptive phase.^40^ Patrizio et al described the same phenomenon in the repair of a subacute tear of the Achilles tendon.^41^ Overall, the BioBrace implant shows promise in augmentation of rotator cuff and other tendon repairs.

## CONCLUSION

In this case, intraoperative findings revealed a rotator cuff tear substantially larger than suggested by preoperative MRI, along with biceps tendon involvement and tearing of the subscapularis tendon. Augmentation with the BioBrace implant provided structural support and a biologically active scaffold to enhance healing. At 1 year postoperatively, MRI demonstrated a heterogeneous tendon signal consistent with excellent graft incorporation and no evidence of retear. This case highlights the potential of biocomposite scaffolds to achieve durable tendon healing in large or underestimated rotator cuff tears.

## Notes

*This article meets the Accreditation Council for Graduate Medical Education and the American Board of Medical Specialties Maintenance of Certification competencies for Patient Care and Medical Knowledge.*
